# Spiritual Care in Advanced Dementia from the Perspective of Health Providers: A Qualitative Systematic Review

**DOI:** 10.1155/2021/9998480

**Published:** 2021-11-24

**Authors:** Lucía Rocío Camacho-Montaño, Jorge Pérez-Corrales, Marta Pérez-de-Heredia-Torres, Ana María Martin-Pérez, Javier Güeita-Rodríguez, Juan Francisco Velarde-García, Domingo Palacios-Ceña

**Affiliations:** ^1^Department of Physical Therapy, Occupational Therapy, Physical Medicine and Rehabilitation, Research Group in Evaluation and Assessment of Capacity, Functionality and Disability of Universidad Rey Juan Carlos (TO+IDI), Universidad Rey Juan Carlos, Alcorcón, Spain; ^2^Department of Physical Therapy, Occupational Therapy, Physical Medicine and Rehabilitation, Research Group of Humanities and Qualitative Research in Health Science of Universidad Rey Juan Carlos (Hum&QRinHS), Universidad Rey Juan Carlos, Alcorcón, Spain; ^3^Department of Nursing, Red Cross College, Universidad Autónoma de Madrid, Gregorio Marañón Sanitary Research Institute (IiSGM), Madrid, Spain

## Abstract

**Background:**

Worldwide, 47 million people suffer from dementia. Despite recognizing the importance of spirituality within dementia care, it is still unclear how this should be integrated into dementia services.

**Aim:**

To explore the perspective of health professionals regarding the spiritual care of people with advanced dementia.

**Methods:**

A qualitative systematic review was performed following the Enhancing Transparency in Reporting the Synthesis of Qualitative Research guidelines for the study design. The inclusion criteria included original articles published from January 2008 to March 2019, using either qualitative or mixed methods. The quality of the articles included was evaluated using the consolidated criteria for reporting qualitative research, Standards for Reporting Qualitative Research, and the Critical Appraisal Skills Programme. Synthesis of findings was performed using thematic analysis.

**Results:**

Twelve studies were included in the review. Seventeen categories were identified, grouped into four themes: (1) the perception of spirituality, including the failure to address the same, (2) the spiritual needs of people with advanced dementia, (3) spiritual needs from health care providers, and (4) addressing spirituality, with the following categories: music, significant activities, among others.

**Conclusions:**

Spirituality is not formally addressed in this population, and professionals do not feel confident enough to be able to integrate spirituality in their care. It is necessary to identify and record the spiritual needs of people with advanced dementia, as well as to design specific care programs.

## 1. Introduction

Worldwide, an estimated 47 million people suffer from dementia [1]. Dementia describes a range of diseases in which there is a decline in memory, thinking, behavior, and the capacity to perform everyday activities [2]. Dementia is a progressive disease, which, in advanced stages leads to severe cognitive impairment, difficulty in verbal communication, and loss of functional capacity [2]. As a result, people with dementia often require institutionalization in nursing homes [3]. Advanced dementia refers to the late stage of the disease and is characterized by immobility or even bed rest, incontinence, total loss of speech, and total care dependency [4]. In these final stages of the disease, communication impairments [4, 5] and difficulty expressing needs [5, 6] hamper the establishment and application of criteria for terminal care in dementia [6, 7], such as access to palliative care [4].

Within palliative care [8], spirituality is one of the least developed dimensions [9]. Spirituality has been defined as “the search for meaning and purpose in life” [10], and it can be understood as an internal force [10–12]. Spirituality can also be strongly related to a feeling of participation within the community [13]. The connection that takes place between the person and the environment, or with his or her inner self, allows for a spiritual experience. The connections that have been highlighted in relation to spirituality include a link with a superior being, with oneself, with others, with nature [10], or with comfort [14]. Each culture or society also has an influence on an individual's sense of connectedness, which in turn it is related to the interconnection between the person, their environment, and their occupation [13].

Previous studies [15, 16] describe how all people have spiritual needs, although their expression and meaning may be different for each person. Promoting the patient's wishes and providing time, space, or meaningful activities have all been identified as ways to support the person's spiritual needs [10]. In accordance with this, it is important to provide people with advanced dementia with something that is meaningful and familiar. Thus, a previous study highlighted the psychosocial needs of people with advanced dementia [6]. The spiritual approach addresses relevant issues of life and death [17], involving the promotion of a connection with oneself and others, as well as attributing meaning to one's life [11]. However, there are barriers among health professionals [10] that influence their ability to integrate spirituality during care, such as lack of safety, and self-efficacy [11]. In addition, professionals believe that it is difficult to apply spirituality in people with dementia [10], or even that people in the last stage of dementia have no spiritual needs [11, 14].

In advanced stages of dementia, it is difficult to manage the transcendental aspects of life [18]. Indeed, it is difficult to identify spiritual needs [15], and these may even be ignored [19]. Previous authors [11, 20] describe the experience of spirituality in people with mild-moderate dementia. These studies show the need to respect the person's preferences and their relationships and highlight the fact that some professionals do not address spirituality [11]. Dalby et al. [20] describe that the search for meaning, integrity in the face of dementia, and ways of connecting and manifesting spiritually are relevant in people with dementia. Furthermore, Higgins [21] and Daly and Fahey-McCarthy [16] explored the role of spirituality in residents with moderate dementia. Thus, faith is part of the residents' identity, and the relationship with God provides a sense of meaning and security in their lives. Similarly, prayer and religious traditions are a source of connection between their past and their present [21].

Health care professionals play a fundamental role in promoting spirituality, yet few actually attempt to promote the same with the patient and/or family [10]. Ødbehr et al. [22] explored how health care professionals address spirituality in people with dementia, considering that this is done subconsciously, through facilitating activities and connections. Likewise, health care professionals pointed out the importance of sensitivity when communicating with residents, especially in those residents who have communication impairments, i.e., by using gestures and body language. Spiritual care is necessary, in the form of including emotional support, offering dignity, comfort, and meaning to patients. Furthermore, there are specific programs such as Namaste Care [4, 23–25]. The Namaste Care program was developed by Simard, in response to evidence of a lack of meaningful engagement in people with advanced dementia [4]. The main aim of this program is to meet their spiritual needs of interaction and participation [25] and is described as a sensory-based approach integrating person-centered nursing care and individualized meaningful activities in a peaceful environment [4].

The question that guided this qualitative review was as follows: What are the perspectives of health care professionals on spirituality and spiritual care in advanced phases of dementia?

The aim of the study was to perform a qualitative systematic review on how healthcare providers of patients in stages of advanced dementia perceive spirituality needs and ways to approach spirituality care.

## 2. Methods

### 2.1. Design

A qualitative systematic review was conducted using thematic synthesis [26], following the recommendations for Enhancing Transparency in Reporting the Synthesis of Qualitative Research (ENTREQ) [27].

### 2.2. Literature Search and Selection

A selective search was conducted between January 2008 and March 2019 via PubMed, CINAHL, EMBASE, Academic Search Complete, JSTOR, ProQuest, PsycARTICLES, PsycINFO, Scopus, Web of Science (WOS), ScienceDirect, Nursing & Allied Health Database, and Google Scholar. The search criteria followed the STARLITE proposal (Table 1), including the description of sampling strategy, type of studies, approaches, range (years), limits, inclusion and exclusion criteria, terms used, electronic sources [28], and search criteria [29, 30]. The *Cochrane Library* and PROSPERO were consulted to confirm the nonexistence of previous systematic reviews on the study topic. See Supplementary file 1: search strategy.

### 2.3. Search Criteria

The search criteria included articles that described and/or analyzed the perspective of health care providers regarding how they understand spirituality, spiritual needs, and care of people in nursing homes over the age of 65 with advanced dementia. Nursing homes were considered as the context for these studies, due to the high rates of institutionalized people with advanced dementia.

### 2.4. Quality Appraisal

The quality of the selected studies was determined using the consolidated criteria for reporting qualitative research (COREQ) [31], the Standards for Reporting Qualitative Research (SRQR) [32], and the Critical Appraisal Skills Programme (CASP) [33]. Both SRQR and COREQ have been considered valid tools for quality appraisal in qualitative research [34], and CASP is used for the synthesis of qualitative evidence in Cochrane reviews [35]. In addition, confidence in findings [36] was assessed by Confidence in Evidence from Reviews of Qualitative research (GRADE-CERQual) [37].

The COREQ is a list of 32 items for qualitative research, including the sampling method, the setting for data collection, the method of data collection, respondent validation of findings, the method of recording data, the description of the derivation of themes, and the inclusion of supporting quotations [31]. The SRQR is a checklist for reporting qualitative research consisting of 21 items which include the article's title and abstract, problem formulation, research question, research design and methods of data collection and analysis, results, interpretation, discussion, and integration [32]. The CASP is a checklist of 10 questions covering validity, the results, and value of qualitative research [33]. The appraisal process was conducted independently by three reviewers (L.R.C-M, J.P-C, and D.P-C), and consensus was required. See Supplementary file 2: quality appraisal CASP; Supplementary file 3: quality appraisal COREQ; and Supplementary file 4: quality appraisal SRQR.

### 2.5. Data Extraction

For data analysis and extraction, the Joanna Briggs Institute-Qualitative Assessment and Review Instrument (JBI-QARI) was used. JBI-QARI is a standard data extraction tool which enables critical appraisal of research describing the characteristics of the studies, incorporating citations, participants, phenomena of interest, settings, methodology, methods, and culture. Secondly, it was carried out extracting findings as the second step of data extraction and first phase of data synthesis. Findings are extracts which are quotes or the author's interpretation of included studies, decoded by the author. The finding is accompanied by an illustration which is a direct quote, fieldwork observations, or other data, from the same writing that reports the finding. There are three grades of credibility: unequivocal: illustrations are beyond rational uncertainty and not opening to questions; credible: illustrations lack clear relation between both illustration and findings and for this reason, open to challenges; unsupported: findings not supported by facts [38]. See Supplementary file 5: JBI-QARI data extraction tool.

### 2.6. Data Synthesis

The process of derivation of themes was inductive. Thematic analysis was used for data synthesis, analyzing the original primary studies line by line and developing codes [27]. The method used was meta-aggregation, by building categories, with at least two outcomes (codes) with similar meanings for each category, and creating themes with synthesized findings from at least two categories. These results were developed by repeatedly reading the included articles. Subsequently, each finding was obtained, along with its respective narratives (verbatim sentence, observations), to analyze the level of congruence between them, with a level of credibility between finding and citation. Unequivocal: findings accompanied by a verbatim sentence that is beyond reasonable uncertainty; credible: findings accompanied by an illustration lacking obvious association with it; unsupported: findings not supported by illustration which was based on author's interpretation. Each finding was reread in depth several times, and a code was assigned based on its meaning; thus, as similarities were found between the findings, the categories were created. In addition, the identified categories were reread several times to determine their coherence and to establish a synthesis of the results [38]. Themes or subthemes were not considered if quotes or author's interpretation is not provided in the original articles. Three reviewers (L.R.C-M, J.P-C, and D.P-C) were involved in coding and analysis. No qualitative software was used on the data. See Supplementary file 5: JBI-QARI data extraction tool.

## 3. Results

### 3.1. Search Outcome

**Figure 1:**
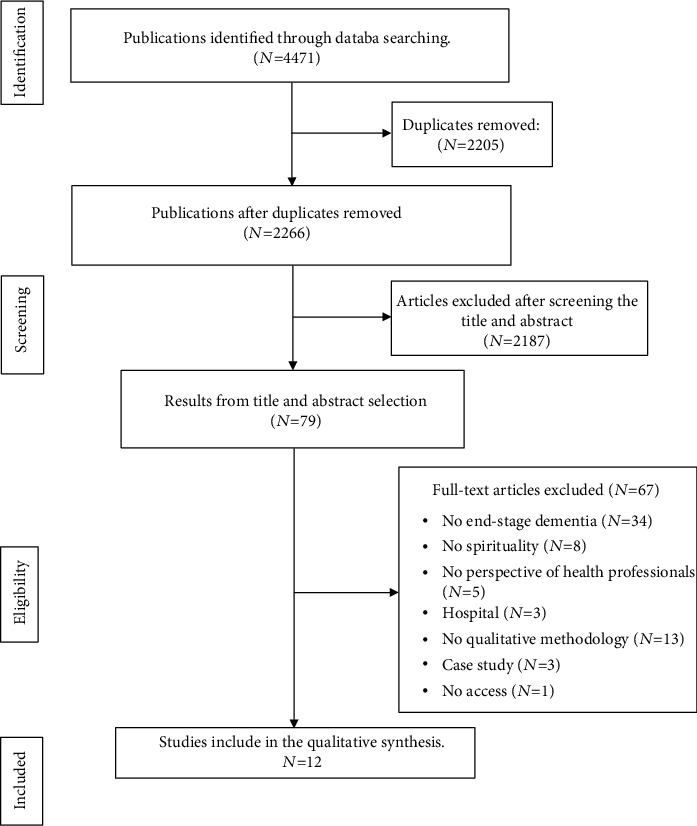
Search diagram.

The initial search retrieved a total of 4471 articles. After removing duplicates, the titles and abstracts of the articles that fulfilled the search and inclusion criteria were evaluated. This reduced the number of articles to 79. After revising the full texts of papers, 12 studies were included. See Figure 1: search diagram.

### 3.2. Study Characteristics

Of the twelve included studies, three were mixed methods and nine were original qualitative studies. Specifically, one study considered the perception of spirituality from the perspective of the health care professionals, three considered spiritual needs, and eight focused on spiritual care. The total number of health professionals who participated was 460. These included nurses [3, 4, 7, 9, 12, 14, 23, 25, 39, 40], social workers [7, 25, 40], nursing assistants [3, 4, 6, 12, 23, 39], physicians [6, 7, 9, 40], and occupational therapists [23, 40]. For more information, see Table 2.

### 3.3. Appraisal Results

All studies covered at least half of the CASP criteria; for this reason, all were included. Moreover, 11 out of 12 studies selected considered more than seven criteria, and the study by Bray et al. [23] covered five items. Only the studies by Bray et al. [23] and Moore et al. [40], respectively, fulfilled 12 and 15 items of the COREQ, whereas the remaining studies covered 16 items or more. Regarding the SRQR, all studies covered at least half of these items.

### 3.4. Results of the Synthesis

The findings were aggregated into seventeen categories, based on which, four synthesized themes were developed: *perception of spirituality*, *spiritual needs of people with advanced dementia*, *spiritual needs of health care professionals*, and *addressing spiritual care in advanced dementia*.

Spiritual care in advanced dementia is based on the perception and the need for spiritual acknowledgment. The importance of training was perceived in order to address spirituality [3, 9, 23, 41], because of the fear of death, among other reasons [3, 39]. Likewise, health care professionals perceive a failure in spiritual care, reporting the lack of a formal approach [3, 7, 9] despite their agreement regarding the needs of spirituality assessments [40, 41]. Further, spiritual conversations [3, 39], music [12, 25, 41], prayer [3, 12, 41], meaningful occupations [4, 9], private spaces [4, 23], and the Namaste Care program [4, 23, 25] have been used as spiritual resources in which health care providers have reported positive responses such as facial expressions [4, 23, 25, 41]. See Table 3: synthesized findings.

#### 3.4.1. Perception of Spirituality

This finding was based on three categories. First, the meaning of spirituality: *“Spirituality isn't always words. Sometimes it's just sitting there holding their hands and letting them know you're present and you're there with them”* [41]*. “It's how you find peace in your soul, what gives you peace in your heart,” “Where someone gets their comfort”* [14].

Secondly, different perspectives arose among nurses regarding the perception of spirituality in advanced dementia: *“I think the end stage of dementia… they are not into the spiritual”*, whereas, according to another nurse: *“It's the exact same. (Older) People with dementia are no different to anyone else”* [14], others agree: *“You may think a person with dementia isn't getting anything out of a worship service. But I don't believe that. I believe if they can still hear … not even that … [because] they can feel that sense, I think”* [41].

The last category described the perception of the failure in the ability to address spirituality. Spiritual care was often not mentioned until prompted, and it was not always addressed as systematically as the accounts of what usual care should entail [7], *“I never really asked [about spiritual needs], sometimes I ask: are you afraid of dying? But I don't ask it at the end of life, but at an earlier stage”* [9], “*We never used to do so well in that [spiritual care] …,”* only covering the basics *“I actually think this home does meet their basic needs … but the rest of the time, people are just left, and I think that that's a tragedy. That to me is a real tragedy of dementia care, of any sort of care, actually just to be left”* [3]; thus, spiritual care is sometimes still neglected [7].

#### 3.4.2. Spiritual Needs of People with Advanced Dementia

This theme was based on four categories, as detailed in eight studies [3, 6, 9, 23, 25, 39–41]. The first category was spiritual assessment; 26.7% of professionals agreed that they should assess religious affiliation and involvement, sources of spiritual support, and the spiritual well-being of patients and their families, whereas 40% strongly agree [40]. Likewise, they felt that these needs should be recognized: *“Not to be afraid to ask them if they'd like to pray ... to take time to pray or go to Mass with them”* [41]. Regarding keeping a record of spiritual needs, these were not mentioned in the care plan and were not discussed with residents [9], neither was religious choice [39].

The need for a familiar environment was highlighted in the next category: *“What I also consider important are … stable structures”* [6], *“Most of them they do want to… be here, in a homely environment”*, likewise *“At the dying stages… this is their home…, so we don't need to send them to the hospital”* [39]. A further category that emerged was the need for person-centered care, perceived by professionals [23], *“They are just treated with the dignity and respect that they deserve*…” [3]. The need for interpersonal relations was identified by professionals as being significant: *“We all need the spirit of somebody else ... human connectedness ... to bring out our spirituality”* [41], especially involving known people: *“What I also consider important are stable relationships”* [6]. In turn, professionals acknowledged the accompaniment needs of residents: *“…She needs us to be there for her…”* [25].

#### 3.4.3. Spiritual Needs of Health Care Professionals

This theme was founded on two categories, as detailed in six studies [3, 9, 23, 25, 39, 41]. The demand for training was considered significant [23]; the professionals need to address spirituality; however, they highlighted the lack of preparation for the same: *“I would like to do it [address spiritual issues], but I would need additional training”* [9]. Less than 19% had ever received training for working with residents with dementia. However, the majority (91%) felt that such care was beneficial, and 65% indicated that additional training would be helpful [41]. The fourth category reaffirms the fear that professionals have regarding the process of death: *“I know a nurse and a couple of our other carers and someone has actually passed away, they freak out…”*; furthermore, there is a lack of confidence in aspects related to death: *“People haven't seen death before and it could have a really adverse reaction on them,”*; furthermore, one of the objectives that are sought is to decrease the fear of death among professionals: *“We try and get all staff to experience the dying process”* [3]. Likewise, discussion of death is considered taboo for the staff: “*It's very scary to say that he [another resident] died …. So some- times we just tell lies or ...change the topic... because … [its] very traumatic for them, ... (it's) not easy to discuss,*”, to the extent that, when residents talk about their own death, this may be considered a weakness: *“One of the residents, she always says - ‘oh I want to die', and I said ‘no, you can't die”* [39].

#### 3.4.4. Addressing Spiritual Care

The seven categories that conform to this theme were described in eight articles [3, 4, 9, 12, 23, 25, 39, 41]. The first category was conversations on spiritual aspects, focusing on whether people wish they could be able to die in the care home: *“not really, because it's a very personal question”* [39], *“You know could you really, just tell somebody not to ring their bell, I mean, what happened to kind, caring, compassion, you know, ‘would you like a cup of tea, how about I sit and have a chat with you for a while'”* [3].

Category number two in this theme was the use of music as a resource “*When she was near to the end, you know, we took her to her room and brought the stereo into the room. I remember she used to like Nat King Cole. And I had put that music on… she was very near to the end… and she held up her head, opened her eyes and smiled…*” [25]. Moreover, music has been applied in people with advanced dementia as they are able to remember the lyrics of songs that are known to them: “*It can make him feel good, reassure him”* [12]. Likewise, music is applied to the next category, religion [3, 12, 41]: “*And often when you hold their hands and pray with them or turn on religious music, their body changes. Their facial expressions often change. Their muscles will relax. Absolutely! I see it”* [41] *“…We found that initially a lot of people weren't seeing the priest or their religion wasn't being addressed”* [3]. Indeed, maintaining a faith community is considered essential *“I think that religious group participation offers a sense of togetherness”, “For some it is the highlight when the priest comes to visit”* [12].

The fourth category was meaningful occupations. Professionals refer that promoting what was significant in the past triggers positive reactions: *“I noticed those residents that were actually responding—you know, those people who cannot actually talk or … this is serious and you give them dolls and they are actually kissing the dolls and talking to them. It's like a real baby for them”* [4]. Attending important events is seen as an informal approach of spirituality [9].

Category number five was related to where the residents were placed, considering the need to place them in a private space: *“Rather than sitting them [the resident] in the corner doing nothing, at least they are in a safe place … a safe and comfortable place”* [4]. Moreover, this was seen as a source of respect: *“*…*when you, sort of, just allocate a particular room and it's just, it's somehow given more respect”* [23].

The sixth category involved the beneficial effects of the Namaste Care program. Thanks to this program, the professionals perceived more interaction between residents, and people were more responsive and communicative [23], making more eye contact, as well as demonstrating a greater intention to relate with others, even with those who have communication problems: *“And you get that relationship… touch builds a relationship between people doesn't it?... So at that moment there is a really good bond between the staff and the resident”* [25]. People were more relaxed, leading to a more positive mood, and were more likely to smile [23]; finding that people were more responsive when a sense of closeness and warmth was conveyed during care: *“There's something that changes within them that you can feel, they can also feel that they're loved”* [4]. In addition, an increased positive mood was reported, as well as decreased passivity: *“They are feeling lighter and they look well. When you look at them, they really look well”* [25].

Category number seven featured other approaches, such as respect for the person's wishes: *“I think it is best to stop offering her food when she retches, to respect this wish”* [9], or the use of reminiscence in dementia care: *“I think that at least the memories of earlier spiritual life are important for people with dementia.”*

## 4. Discussion

No specific research has explored health professionals' perceptions regarding how to address the spirituality of people with advanced dementia. Overall, the results show that there is a failure in spiritual care, especially in advanced dementia [3, 7, 9] despite the agreement concerning the need for this type of care [40, 41]. The failure to address spiritual care can stem from the lack of recognition of an individual's spirituality needs, which in turn may be due to the lack of preparation and training of health care providers [3, 9, 23, 41]. Nonetheless, a review by Kevern [42] considers that spirituality should be promoted until the last stage of dementia. In turn, professionals feel that it is necessary to make note of the person's spiritual needs [40], as well as their spiritual development [15, 19] and their practices and traditions. Nonetheless, the assessment of spiritual needs tends to be ignored [15], which results, as our results reveal, in a lack of treatment for spiritual and religious needs in the care plan of people with dementia [9, 39].

Perrar et al. [5] describe how people with severe dementia need to meet their needs for interaction, participation, and privacy and to die in peace and in their place of choice. In addition, it is necessary, especially in dementia, to provide comfort and respect for their spirituality [14]. In order to do so, it is essential to promote the expression of beliefs and values, maintain connections [15], and preserve their dignity [5, 15, 19], their identity, and the meaning of life [15, 19], as well as to continue being part of their community, and communicating with God [19]. Health professionals identify numerous needs, such as religion [40, 41], treating people with dignity and respect [4, 23], and enhancing social relationships [41], especially with people they know [6]. In turn, professionals feel the desire to respond to this need [25].

Previous studies show how spiritual needs can be addressed by facilitating a sense of familiarity, preserving meaningfulness, connections to community [15], promoting emotions associated with music, objects, people, and reminiscence [19]. However, our results show that professionals do not tend to formally work towards and encourage the spiritual approach [3, 4, 7, 9]. Nonetheless, professionals try to help residents die in peace, complete their lives, or maintain relationships with their loved ones [9].

In addition, various forms of spiritual assistance have been identified, such as music [12, 41], religion [3, 12, 41], meaningful occupations [4, 9], a private environment [4, 23], respect for their wishes [9], and reminiscence [12], making residents feel that one is there for them [41], regardless of their communication skills [12], thanks to close contact and use of empowering words [41]. Thus, Ødbehr et al. [22] describe how nurses maintain that physical contacts, such as holding hands, giving hugs, and looking into a person's eyes, are all part of the spiritual approach. Other studies on the perspective of health professionals have identified activities as a manner of addressing spirituality. What is more, professionals have recognized occupational therapists as professionals who are able to address spiritual needs by involving people with dementia in valued and meaningful activities [10]. However, this systematic review only found two studies that included occupational therapists [23, 40]. Kielsgaard et al. [43] highlighted that there are few opportunities for engagement in activities in dementia care, especially in advanced stages of dementia.

Facilitating participation through familiar prayers and songs at later stages promotes self-efficacy [44]. However, religion is not being addressed, as only 3% of people with dementia attend religious services [41], nor do they receive visits from priests [3]. In this vein, other studies on dementia report that nurses are not open to praying with residents even though this has been perceived as a spiritual approach [22]; however, nurses acknowledge that religion continues to be important [20, 21].

Occupations have been used as part of a spiritual approach [3, 4, 9, 12, 41]. This is in line with a study by Ødbehr et al. [22] in which professionals claim that spirituality can be addressed through meaningful activities. To this end, by facilitating activities, professionals seek to make residents' lives as meaningful as possible. In addition, Han et al. [45] argue that participation in meaningful occupations is an important motivation for people with dementia, keeping them connected to themselves, to others, and to the environment. Meaningfulness has been related to spirituality because definitions of both are similar: “connectedness in being and doing”; according to this, it is known that professionals must provide familiar, symbolic, and emotional care to people with advanced dementia [46].

Dementia should not hinder the expression of spirituality [10], as the emotional bond remains intact in the face of cognitive impairment [19]. Most studies only include people with mild dementia who can still express themselves; however, residents with advanced dementia should also be considered [6, 47]. Currently, due to lack of research, it is questionable whether these needs are still being met at advanced stages of the disease, as well as whether professionals are sensitive in their ability to detect spirituality [6].

Care home staff report that people with advanced dementia prefer to die in familiar settings, such as nursing homes [39], although these settings are often viewed negatively due to the lack of professional training [3, 9, 23, 41]. In matters of spirituality, professionals also display a lack of confidence [10, 14], due to a lack of adequate training in spiritual approaches [11].

This paper provides an overview of current evidence on the importance of spirituality in advanced dementia. Our results suggest that meaningful occupations are a significant way to trigger positive responses in the last stage of dementia [4, 9], such as body changes [4, 12, 41] and facial expressions like smiling, making more eye contact, or being more responsive and communicative [4, 23, 25]. In line with this, other authors have recognized that the emotional bond remains intact in even severe cognitive impairment [19]. Meaningful activities are related to the emotional connection and provide a sense of connectedness. Given that this is essential to the occupational therapy process, occupational therapists should focus on the purposefulness and meaningfulness of providing activities in dementia care [48]. This requires good observation skills to be sensitive to the different means of communication imposed by severe cognitive impairment [6]. There are other sources for obtaining information, including life history or family interviews [6, 47]. In this sense, various activities have been identified as forms of spiritual assistance in people with advanced dementia, such as music [12, 41], religion [3, 12, 41], or a private environment [4, 23].

### 4.1. Implications for Future Research and Clinical Practice

Therefore, it is important to develop further studies that focus on understanding the spirituality of people in the advanced stages of dementia, as well as for training health care providers due to their feelings about the lack of preparation for the same. Future research should be conducted to assess the beneficial effect of spiritual conversations, meaningful activities, music, religion, or the Namaste Care program in residents with advanced dementia. These results can help professionals who care for residents with dementia to be sensitive to the spiritual and religious needs of residents. Furthermore, considering that occupational therapists focus on meaningful activities, and these have been considered a form of spiritual approach in dementia care, occupational therapists should be involved in the design of specific, valid, and reliable programs for addressing spiritual care through meaningful occupations such as music or religion. Thus, future studies are required in this line for individuals with advanced dementia.

### 4.2. Strengths and Limitations

The main strength of this review is the application of international recommendations for several aspects, including the study design (ENTREQ), the criteria used in the literature search (STARLITE), the assessment of its internal structure (COREQ, SRQR, and CASP), and the data extraction methods (JBI-QARI). Furthermore, there are several limitations to this review including the exclusion of studies in languages other than English and Spanish. Not all studies included information on the dementia severity of the people recruited; therefore, we included all papers referring to dementia, its staging or severity, and which included patients in the advanced stage of the disease. Despite these limitations, the present study provides the basis for a spiritual approach to advanced dementia, and it may contribute to develop intervention programs for occupational therapists to enable the evaluation and monitoring of meaningful activities that enhance the spirituality of people with advanced dementia.

## 5. Conclusions

The detection of the spiritual needs of people with dementia is limited, and the ability for health professionals to address and manage spirituality in nursing homes is scarce. Thus, spirituality is not being formally addressed by the institutions, nor do the professionals feel confident in their ability to address these issues. Certain initiatives, such as Namaste Care, may be an alternative in advanced dementia; however, these measures are only used on a local or regional level, and not internationally implemented. Our findings highlight a need to establish programs for nursing homes to enable the evaluation and monitoring of activities that develop and enhance the spirituality of people with advanced dementia.

## Figures and Tables

**Table 1:** STARLITE proposal [28].

Sampling strategy	Selective sampling strategy
Type of studies	Qualitative research Studies using mixed methods were included if the qualitative data could be extracted
Approaches	Electronic searching
Range of years	January 2008-March 2019
Limits	English or Spanish language years: January 2008-March 2019 Type of studies: research articles
Inclusion and exclusions	The study inclusion criteria were original research, qualitative studies, or studies using mixed methods (if the qualitative data could be extracted), written in English or Spanish, from January 2008 to March 2019. Studies were excluded if they were not primary research or qualitative methods.
Terms used	Medical Subject Headings (MESH): “Dementia” “Alzheimer Disease” “Spirituality” “Terminal Care” “Interview” “Focus group” “Surveys and Questionnaires”. Free term: “Advanced Dementia” “Severe Dementia” “End-stage Dementia” “Advanced Alzheimer” “Severe Alzheimer” “End-stage Alzheimer” “Spirit∗” “Namaste Care” “experience” “perception” “perspective” “view” “Palliative Care”.
Electronic sources	Cochrane, Prospero, EMBASE, Pubmed/Medline, Cumulative Index to Nursing and Allied Health Literature (CINAHL), Academic Search Complete, JSTOR, ProQuest, PsycARTICLES, PsycINFO, Scopus, Web of Science (WOS), ScienceDirect, Nursing & Allied Health Database, Google Scholar

**Table 2:** Characteristics of studies included.

Reference	Purpose	Design	Sampling strategy	Sample	Data collection methods	Data analysis
Bray et al., 2019 (UK) [23]	To determine an intervention based on the principles of Namaste Care, learning from the experience of others who are implementing this in nursing homes	Mixed methods: sequential investigation	Not specified	Survey: 100 participants Interview: 13 participants (6 managers, 1 assistant nurse, 1 nurse, 1 consultant, 1 dementia care specialist, 1 occupational therapist, 3 trainers)	Survey/in-depth interviews	Quantitative study: not specified Qualitative study: thematic
Chang et al., 2019 (Wales, UK) [4]	To provide comprehensive and sustainable care, honoring and respecting the person	A qualitative study from a larger mixed-method research	Purposive sampling	20 (12 nurses, 8 assistants in nursing)	Focus groups (semistructured interview schedule)	Thematic
Gijsberts et al., 2013 (Dutch) [9]	To explore both the needs as well as the approach of spirituality, including dementia and the contribution of caregivers in the spiritual needs of residents	Ethnographic	Not specified	8 (4 nurses, 3 physicians, 1 spiritual counsellor)	Formal interviews	Inductive thematic
Keenan & Kirwan, 2018 (Ireland) [14]	To explore the perception of spirituality of nurses in older people living with dementia	A qualitative descriptive research approach	Purposive sampling	8 nurses	Interviews (interview schedule informed)	Thematic
Kupeli et al., 2016 (London, UK) [3]	To identify the factors, as well as their influence on the results on end of life care in advanced dementia	Qualitative study (realist approach)	Purposive sampling	14 (4 nurses, 3 health care assistants, 3 managers, 2 joint commissionings, 1 occupational therapist, 1 mental health professional)	Interactive interviews	Thematic
Livingston et al., 2012 (London, UK) [39]	To examine barriers and facilitators for the nursing home staff, to improve the approach for end of life in people with dementia	Qualitative methodology	Not specified	58 (30 care workers, 20 nurses, 8 senior careers)	Interviews (interview guide)	Broadly thematic content-analytic approach
Moore et al., 2019 (London, UK) [40]	To explore the practice and the role played by the services for helping to prepare end of life care	Mixed methods: cross-sectional survey and interview study	Not specified	Quantitative study: 45 survey participants (39 nurses, 2 occupational therapists, 2 social workers, 1 doctor, 1 psychologist) Qualitative study: 12 participants (1 team leader, 3 consultant psychiatrists, 5 nurses, 1 care coordinator, 1 occupational therapist, 1 support worker)	Survey/semistructured interviews (interview guide)	Quantitative study: standard descriptive statistics Qualitative study: inductive analysis
Powers & Watson, 2011 (New York, US) [41]	To understand spirituality; examine the perception of health professionals as well as the family members on the spiritual approach; to analyze the resources for an assessment and intervention of the spiritual needs	Mixed methods	Quantitative study: random stratified sample Qualitative study: purposefully selected	Quantitative study: a survey of a 21% sample of staff. A 38% reply rate was accomplished Qualitative study: 66 nursing home staff (nurses and nursing assistants, social workers, recreation and physical therapists)	Quantitative data collection: surveys Qualitative data collection: semistructured interviews	Quantitative study: descriptive statistics Qualitative study: thematic
Schmidt et al., 2018 (Germany) [6]	To identify the needs of advanced dementia in the final phase and to explore the relevant aspects which must be covered	Grounded theory	Convenience sampling	42 (33 caregivers, 3 physicians, 3 housekeepers, 2 others, 1 physician)	Semistructured group discussion/interview individual	Comparative method analysis
Stacpoole et al., 2017 (UK) [25]	To establish whether the Namaste Care program can be implemented in nursing homes in the United Kingdom and what effect this has on the quality of life of residents with advanced dementia and their family members	A qualitative study (organizational action research) from a larger mixed-method research	Not specified	Focus group preimplementation: 40 (9 nurses, 28 care workers, 3 activities coordinators) Focus group post: 31 (7 nurses, 22 care workers, 2 activity coordinators) 5 managers interviewed	Focus groups/semistructured interviews	Grounded theory analysis
Toivonen et al., 2018 (Finland) [12]	To describe the experiences of nurses in their approach on spirituality of older people living with dementia	Heideggerian hermeneutic phenomenology	Purposive sampling	17 female nurses; (9 registered nurses, 8 assistant nurses)	Unstructured interviews	Inductive content
van der Steen et al., 2017 (Netherlands, UK, US, Belgium, Israel) [7]	To understand the needs in the development of PC needs in terminal stages of dementia	Qualitative methodology	Not specified	11 physicians, 10 nurses, 1 social worker, 3 project leaders	Focus groups/interviews (interview guide)	Thematic

**Table 3:** Synthesized findings.

Themes	Categories
Perception of spirituality	(i) Spirituality [14, 41] (ii) Spirituality in end stage of dementia [14, 41] (iii) Unsuccessful spiritual approach [3, 7, 9]
Spiritual needs of people with advanced dementia	(i) Spiritual care plan record [9, 39, 40] (ii) Familiar environment [6, 39] (iii) Need for person-centered care [3, 23] (iv) Interactions [6, 25, 41]
Spiritual needs of health care professionals	(i) Additional training [3, 9, 23, 41] (ii) Fear of death [3, 39]
Spiritual care	(i) Conversations [3, 39] (ii) Music [12, 25, 41] (iii) Religion [3, 12, 41] (iv) Meaningful occupations [4, 9] (v) Private space [4, 23] (vi) Namaste Care effects [4, 23, 25] (vii) Other approaches [9, 12]

## Additional Points

The following references mentioned in this article included qualitative systematic review articles: Kupeli et al. (2016), Chang et al. (2018), Schmidt et al. (2017), van der Steen et al. (2017), Gijsberts et al. (2018), Toivonen et al. (2017), Keenan & Kirwan (2018), Bray et al. (2018), Stacpoole et al. (2017), Livingston et al. (2011), Moore et al. (2018), and Powers & Watson (2011).

## Supplementary Materials

Supplementary Materials Supplementary files 1, 2, 3, specifically, Tables S2, S3, and S4 show the quality appraisal analysis of included studies. In addition, the extraction procedure is detailed in supplementary file 5.
