# Supplementary material for: Spiritual Care in Advanced Dementia from the Perspective of Health Providers: A Qualitative Systematic Review
Source: Occup Ther Int. 2021 Nov 24;2021:9998480. doi: 10.1155/2021/9998480 (PMC8635933; doi:10.1155/2021/9998480)
Supplement: Supplementary Materials — Supplementary files 1, 2, 3, specifically, Tables S2, S3, and S4 show the quality appraisal analysis of included studies. In addition, the extraction procedure is detailed in supplementary file 5. [file 9998480.f1.docx]

| Search strategy | | Data bases |
| --- | --- | --- |
| 1 | (Dementia OR“Alzheimer Disease”) AND“Terminal Care” AND Spirituality | Cochrane, Prospero, EMBASE, Pubmed/Medline, Cumulative Index to Nursing and Allied Health Literature (CINAHL), Academic Search Complete, JSTOR, ProQuest, PsycARTICLES, PsycINFO, Scopus, Web of Science (WOS), Sciencedirect, Nursing & Allied Health Database, Google Scholar |
| 2 | (Dementia OR“Alzheimer Disease”) AND (interview OR“Focus group” OR“Surveys and questionnaires”) AND Spirituality |  |
| 3 | (“Advanced Dementia” OR“Severe Dementia” OR“End-stage Dementia” OR“Advanced Alzheimer” OR“Severe Alzheimer” OR“End-stage Alzheimer”) AND spirit* |  |
| 4 | (“Advanced Dementia” OR“Severe Dementia” OR“End-stage Dementia” OR“Advanced Alzheimer” OR“severe Alzheimer” OR“end-stage Alzheimer”) AND spirit* AND (experience OR perception OR perspective OR view) |  |
| 5 | (“Advanced dementia” OR“severe dementia” OR“end-stage dementia” OR“Advanced Alzheimer” OR“severe Alzheimer” OR“end-stage Alzheimer”) AND “Namaste Care” AND (experience OR perception OR perspective OR view) |  |
| 6 | (“Advanced Dementia” OR“Severe Dementia” OR“End-stage Dementia” OR“Advanced Alzheimer” OR“severe Alzheimer” OR“end-stage Alzheimer”) AND (“End of life care” OR“Palliative care” OR“Terminal care”) AND spirit* |  |

**Supplementary file 1. Search strategy**

**Supplementary file 2. Quality appraisal CASP**

Eleven articles covered more of 7 items [3,4,6,7,9,12,14,25,39,40,41], except for an article [23]. Item 6 “Relationship between researcher and participants”, is only considered by the study by Schmidt et al. [6]. All articles covered the item 1 “Clear aim of the research” and the item 7 “Ethical issues”.

Table S2. Quality appraisal CASP [33].

| Reference | 1 | 2 | 3 | 4 | 5 | 6 | 7 | 8 | 9 | 10 | TOTAL |
| --- | --- | --- | --- | --- | --- | --- | --- | --- | --- | --- | --- |
| Bray et al. [23] | • |  |  | • | • |  | • |  |  | • | 5 |
| Chang et al. [4] | • | • |  | • | • |  | • | • | • | • | 8 |
| Gijsberts et al. [9] | • | • | • |  | • |  | • | • | • | • | 8 |
| Keenan & Kirwan [14] | • | • | • | • | • |  | • | • | • |  | 8 |
| Kupeli et al. [3] | • | • | • | • |  |  | • | • | • | • | 8 |
| Livingston et al. [39] | • | • |  | • | • |  | • | • | • | • | 8 |
| Moore et al. [40] | • |  | • | • | • |  | • | • | • | • | 8 |
| Powers & Watson [41] | • | • |  | • | • |  | • | • | • | • | 8 |
| Schmidt et al. [6] | • | • | • | • | • | • | • | • | • |  | 9 |
| Stacpoole et al. [25] | • | • | • |  | • |  | • | • | • | • | 8 |
| Toivonen et al. [12] | • | • | • | • | • |  | • | • | • | • | 9 |
| Van der Steen et al. [7] | • | • | • | • | • |  | • | • | • | • | 9 |

Notes: 1: clear aim of the research; 2: appropriate qualitative methodology; 3: appropriate research design to address the aims; 4: appropriate recruitment strategy; 5: appropriate data collected; 6: the relationship between researcher and participants has been adequately considered; 7: ethical issues have been taken into consideration; 8: rigorous data analysis; 9: clear statement of findings; 10: valuable research.

**Supplementary file 3. Quality appraisal COREQ**

Ten articles were included which fulfilled half or more criteria [3,4,6,7,9,12,14,25,39,41]. None of the articles considered item 4 “Gender” nor item 13 “Non-participation”.

Table S3. Quality appraisal COREQ [31].

| Reference | 1 | 2 | 3 | 4 | 5 | 6 | 7 | 8 | 9 | 10 | 11 | 12 | 13 | 14 | 15 | 16 | 17 | 18 | 19 | 20 | 21 | 22 | 23 | 24 | 25 | 26 | 27 | 28 | 29 | 30 | 31 | 32 | TOTAL |
| --- | --- | --- | --- | --- | --- | --- | --- | --- | --- | --- | --- | --- | --- | --- | --- | --- | --- | --- | --- | --- | --- | --- | --- | --- | --- | --- | --- | --- | --- | --- | --- | --- | --- |
| Bray et al. [23] |  |  | • |  |  |  | • |  |  |  | • | • |  | • |  | • |  |  | • |  | • |  |  | • |  |  |  |  | • | • | • |  | 12 |
| Chang et al. [4] |  | • | • |  |  |  | • |  |  | • | • | • |  | • |  |  | • |  | • |  | • |  |  |  | • | • |  |  | • | • | • | • | 16 |
| Gijsberts et al. [9] | • | • | • |  |  |  | • |  | • |  |  | • |  | • | • |  |  |  | • | • |  |  |  | • | • | • |  |  | • | • | • | • | 17 |
| Keenan & Kirwan [14] |  |  | • |  |  |  | • |  | • | • | • | • |  | • |  | • | • |  | • | • | • |  |  |  | • | • |  |  | • | • | • | • | 18 |
| Kupeli et al. [3] | • |  | • |  |  |  |  |  | • | • | • | • |  | • |  | • | • |  | • |  | • |  |  | • | • | • | • |  | • | • | • | • | 19 |
| Livingston et al. [39] |  |  | • |  |  |  | • |  |  |  | • | • |  |  |  | • | • |  | • |  | • | • |  | • | • | • | • |  | • | • | • |  | 16 |
| Moore et al. [40] | • |  | • |  |  |  |  |  |  |  | • | • |  | • |  | • | • |  | • |  |  |  |  | • | • | • | • |  | • | • | • |  | 15 |
| Powers & Watson (2011) | • |  | • |  | • | • | • | • |  | • | • | • |  | • |  | • | • |  | • | • |  |  |  |  | • | • |  |  | • | • | • | • | 20 |
| Schmidt et al. (2018) | • |  | • |  |  |  | • |  | • | • | • | • |  | • |  | • | • |  | • | • | • | • |  | • | • | • | • |  | • | • | • | • | 22 |
| Stacpoole et al. (2017) | • |  | • |  |  |  |  |  | • |  | • | • |  | • |  |  | • |  | • | • | • |  |  | • | • | • |  |  | • | • | • | • | 17 |
| Toivonen et al. (2018) | • | • | • |  |  |  | • |  | • | • | • | • |  | • |  | • | • | • | • | • | • | • |  |  | • | • |  |  | • | • | • | • | 22 |
| Van der Steen et al. (2017) | • |  | • |  |  |  |  |  |  |  | • | • |  | • | • | • | • |  | • | • |  |  | • | • | • | • |  | • | • | • | • |  | 18 |

Notes: 1: Interviewer/facilitator; 2: Credentials; 3: Occupation; 4: Gender; 5: Experience and training; 6: Relationship established; 7: Participant knowledge of the interviewer; 8: Interviewer characteristics; 9: Methodological orientation and theory; 10: Sampling; 11: Method of approach; 12: Sample size; 13: Non-participation; 14: Setting of data collection; 15: Presence of non-participants; 16: Description of sample; 17: Interview guide; 18: Repeat interviews; 19: Audio/visual recording; 20: Field notes; 21: Duration; 22: Data saturation; 23: Transcripts returned; 24: Number of data coders; 25: Description of the coding tree; 26: Derivation of themes; 27: Software; 28: Participant checking; 29: Quotations presented; 30: Data and findings consistent; 31: Clarity of major themes; 32: Clarity of minor themes.

**Supplementary file 4. Quality appraisal SRQR**

The three criteria (S1, S5, S6), are those which were least included. Only one study fulfilled criterion S6 “Researcher characteristics” [41]. Only five studied fulfilled criterion S5 “Qualitative approach” [6,9,12,14,25].

Table S4. Quality appraisal SRQR [32].

| Reference | S1 | S2 | S3 | S4 | S5 | S6 | S7 | S8 | S9 | S10 | S11 | S12 | S13 | S14 | S15 | S16 | S17 | S18 | S19 | S20 | S21 | TOTAL |
| --- | --- | --- | --- | --- | --- | --- | --- | --- | --- | --- | --- | --- | --- | --- | --- | --- | --- | --- | --- | --- | --- | --- |
| Bray et al. [23] |  |  | • | • |  |  | • |  | • |  | • | • | • | • | • | • | • | • |  | • | • | 14 |
| Chang et al. [4] |  | • | • | • |  |  | • | • | • |  | • | • | • | • |  | • | • | • | • | • | • | 16 |
| Gijsberts et al. [9] | • | • | • | • | • |  | • |  | • | • | • | • | • | • | • | • | • | • | • | • | • | 19 |
| Keenan & Kirwan [14] |  |  | • | • | • |  | • | • | • |  | • | • | • | • |  | • | • | • | • | • |  | 15 |
| Kupeli et al. [3] |  | • | • | • |  |  | • | • | • | • | • | • | • | • | • | • | • | • | • | • | • | 18 |
| Livingston et al. [39] | • | • | • | • |  |  | • |  | • |  | • | • | • | • | • | • | • | • | • | • | • | 17 |
| Moore et al. [40] |  | • | • | • |  |  | • |  | • | • | • | • | • | • | • | • | • | • | • |  | • | 16 |
| Powers & Watson (2011) |  |  | • | • |  | • | • | • | • |  | • | • | • | • |  | • | • | • |  |  | • | 14 |
| Schmidt et al. (2018) | • | • | • | • | • |  | • | • | • | • | • | • | • | • | • | • | • | • | • | • | • | 20 |
| Stacpoole et al. (2017) | • | • | • | • | • |  | • |  | • | • | • | • | • | • | • | • | • | • | • | • |  | 18 |
| Toivonen et al. (2018) | • | • | • | • | • |  | • | • | • | • | • | • | • | • | • | • | • | • | • |  | • | 19 |
| Van der Steen et al. (2017) | • | • | • | • |  |  | • |  | • | • | • | • | • | • | • | • | • | • | • | • | • | 18 |

Notes: S1: Tittle; S2: Abstract; S3: Problem formulation; S4: Purpose; S5: Qualitative approach and research paradigm; S6: Researcher characteristics; S7: Context; S8: Sampling strategy; S9: Ethical issues; S10: Data collection methods; S11: Data collection instruments; S12: Units of study; S13: Data processing; S14: Data analysis; S15 Techniques to enhance trustworthiness; S16: Synthesis and interpretation; S17: Links to empirical data; S18: Integration with prior work, implications, transferability, and contribution(s); S19: Limitations; S20: Conflicts of interest; S21: Funding

**Supplementary file 5. S5.** Joanna Briggs Institute-Qualitative Assessment and Review Instrument (JBI-QARI) Data Extraction Tool [38].

The results are presented in 12 tables. In each table, the narratives (illustrations) appear, with their page number (p), showing the evidence of the results. Each table is accompanied by the reference from where it was obtained: **Table S5.1.** Bray et al. [23], **Table S5.2.** Chang et al. [4], **Table S5.3.** Gijsberts et al. [9], **Table S5.4.** Keenan & Kirwan [14], **Table S5.5.** Kupeli et al. [3], **Table S5.6.** Livingston et al. [39], **Table S5.7.** Moore et al. [40], **Table S5.8.** Powers & Watson [41],**Table S5.9.** Schmidt et al. [6] , **Table S5.10.** Stacpoole et al. [7], **Table S5.11.** Toivonen et al. [12], **Table S5.12.** Van der Steen et al. [7]. The evidence is allocated in: unsupported, credible e unequivocal [38]:

- Unequivocal: findings accompanied by an illustration that is beyond reasonable uncertainty.
- Credible: findings accompanied by an illustration lacking obvious association with it.
- Unsupported: findings not supported by ilustration.

**Table S5.1.**  Findings from Bray et al. [23]

| **FINDINGS** | **ILUSTRATION** | **EVIDENCE** |
| --- | --- | --- |
| Training required | Training was regarded as highly important *(p.24)* | UNSUPPORTED.  It was based on author’s interpretation |
| Protected space | *“Something very strange happens around when you, sort of, just allocate a particular room and it's just, it's somehow given more respect” (p.25)* | UNEQUIVOCAL |
| Namaste Care needs person-centred care | The approach was perceived as needing to be underpinned by a working knowledge of person-centred care *(p.26)* | UNSUPPORTED. It was based on author’s interpretation |
| Namaste Care Effects | This included reports that it had a relaxing and calming influence, made people more settled, less agitated, led to a more positive mood and more likely to smile *(p.26)* | UNSUPPORTED. It was based on author’s interpretation |
| Namaste Care Effects | From a social perspective, respondents reported a greater sense of inclusion, more interaction between residents, and people were more responsive and communicative *(p.26)* | UNSUPPORTED. It was based on author’s interpretation |

**Table S5.2**. Findings from Chang et al. [4]

| **FINDINGS** | **ILLUSTRATION** | **EVIDENCE** |
| --- | --- | --- |
| Protected space | *“Rather than sitting them [the resident] in the corner doing nothing, at least they are in a safe place … a safe and comfortable place” (p.4)* | UNEQUIVOCAL |
| Meaning in the past | *“I noticed those residents that were actually responding - you know, those people who cannot actually talk or … this is serious and you give them dolls and they are actually kissing the dolls and talking to them. It's like a real baby for them” (p.4)* | CREDIBLE |
| Namaste Care Effects | *“There's something changing within them that you can feel, that they're really—they can also feel that they're loved” (RN Lucy) (p.5)* | UNEQUIVOCAL |
| More communicative | *“There's something changing within them that you can feel, that they're really … they can also feel that they're loved” (RN Leanne) (p.5)* | UNSUPPORTED |

**Table S5.3.** Findings from Gijsberts et al. [9]

| **FINDINGS** | **ILLUSTRATION** | **EVIDENCE** |
| --- | --- | --- |
| Spiritual needs were not detailed | Spiritual needs were not mentioned in the care plan and were not discussed with residents during bedside consultation or in multidisciplinary meetings. *(p.681)* | UNSUPPORTED. It was based on author’s interpretation |
| Spirituality was not approached | *“I never really asked [about spiritual needs], sometimes I ask: are you afraid of dying? But I don’t ask it at the end of life, but at an earlier stage.” (p. 681)* | UNEQUIVOCAL |
| Training required | *“I would like to do it [address spiritual issues], but I would need additional training on how you address these issues, or what kind of questions” (p. 681)* | UNEQUIVOCAL |
| Informal care of meaningful events | *“So the moment I saw the daughter in tears when she said: ‘I have no one to escort my mother to my wedding, what am I to do?’ I thought: I cannot leave it at this, and I said: ‘I will escort your mother to your wedding.” (p.682)* | UNEQUIVOCAL |
| Respect wishes | *“I think it is best to stop offering her food when she retches, to respect this wish.” (p.683)* | UNEQUIVOCAL |

**Table S5.4**. Findings from Keenan & Kirwan [14]

| **FINDINGS** | **ILLUSTRATION** | **EVIDENCE** |
| --- | --- | --- |
| Meaning of spirituality | *“It’s how you find peace in your soul, what gives you peace in your heart” (p.5)* | CREDIBLE |
| Meaning of spirituality | *“Where someone gets their comfort” (p.5)* | UNEQUIVOCAL |
| Perception of spirituality in advance stage of dementia | *“I think the end stage of dementia… they are not into the spiritual” (p.5)* | UNEQUIVOCAL |
| Perception of spirituality in dementia | *“It’s the exact same. (Older) People with Dementia are no different to anyone else” (p.5)* | CREDIBLE |

**Table S5.5**. Findings from Kupeli et al. [3]

| **FINDINGS** | **ILLUSTRATION** | **EVIDENCE** |
| --- | --- | --- |
| Training required due to fear of death | *“We try and get all staff to experience the dying process” (p.6)* | UNEQUIVOCAL |
| The neccesity of conversation | *“You know could you really, just tell somebody not to ring their bell, I mean, what happened to kind, caring, compassion, you know, ‘would you like a cup of tea, how about I sit and have a chat with you for a while’” (p.7)* | UNEQUIVOCAL |
| Basic approach | *“I actually think this home does meet their basic needs - they eat well, they look well… they look nice if you go and see them, their rooms are nice and tidy - but the rest of the time, people are just left and I think that that’s a tragedy. That to me is a real tragedy of dementia care, of any sort of care, actually, just to be left” (p.8)* | UNEQUIVOCAL |
| Person-centered care is needed | *“They are just treated with the dignity and respect that they deserve, and that they’re seen as individuals really…” (p.8)* | UNEQUIVOCAL |
| Lack of confidence in issues related to death | *“People haven’t seen death before and it could have a really adverse reaction on them” (p.7)* | UNEQUIVOCAL |
| Fear of death | *“I know a nurse and a couple of our other carers and someone has actually passed away, they freak out, they freak out! …” (p.9)* | UNEQUIVOCAL |
| Unsuccessful spiritual approach | *“We never used to do so well in that [spiritual care] and that’s one of the things we identified doing after death, significant event analysis…” (p.10)* | UNEQUIVOCAL |
| Unsuccessful religion approach | *“…We found that initially a lot of people weren’t seeing the priest or their religion wasn’t being addressed” (p.10)* | UNEQUIVOCAL |

**Table S5.6**. Findings from Livingston et al. [39]

| **FINDINGS** | **ILLUSTRATION** | **EVIDENCE** |
| --- | --- | --- |
| Religion was not considered in the care plan | Some staff felt (incorrectly) that the lack of plans reflected the residents’ cultural and religious choice. *(p.646)* | UNSUPPORTED. It was based on author’s interpretation |
| Fear of death | *“It’s very scary to say that he [another resident] died …. So some- times we just tell lies or ...change the topic... because … [its] very traumatic for them, ... (it’s) not easy to discuss.” (p.648)* | UNEQUIVOCAL |
| Expression of wish death | *“A resident, she always say - ‘oh I want to die’, and I said ‘no, you can’t die’...” (p.648).* |  |
| Desire of be in a family environment | *“Most of them they do want to… be here, in a homely environment” (p.648).* | UNEQUIVOCAL |
| Desire of die in a family environment | *“At the dying stages… this their home…, so we don’t need to send them to the hospital” (p.648).* | UNEQUIVOCAL |
| End of life conversations | *Wishing people to be able to die in the care home: “really no, it’s because it’s a very personal question” (p.648).* | UNEQUIVOCAL |

**Table S5.7**. Findings from Moore et al. [40]

| **FINDINGS** | **ILLUSTRATION** | **EVIDENCE** |
| --- | --- | --- |
| Spiritual assessment | *Professionals should assess religious affiliation and involvement, sources of spiritual support, and the spiritual well‐being of patients and their families (26,7% agree and 40% strongly agree) (p.5)* | UNEQUIVOCAL |

**Table S5.8**. Findings Powers &Watson [41]

| **FINDINGS** | **ILLUSTRATION** | **EVIDENCE** |
| --- | --- | --- |
| Perception of dementia | *“You look for those little clues. For instance, you may think a person with dementia isn’t getting anything out of a worship service. But I don’t believe that. I believe if they can still hear … not even that … [because] they can feel that sense, I think” (p.67)* | UNEQUIVOCAL |
| Effects of spiritual care | *“**And often* *when you hold their hands and pray with them or turn on religious music, their body changes. Their facial expressions often change. Their muscles will relax. Absolutely! I see it.” (p.67)* | UNEQUIVOCAL |
| Spiritual needs | *“Not to be afraid to ask them if they’d like to pray ... to take time to pray or go to Mass with them” (p.67)* | UNEQUIVOCAL |
| Meaning of spirituality | *“Spirituality isn’t always words. Sometimes it’s just sitting there holding their hands and letting them know you’re present and you’re there with them” (p.71)* | UNEQUIVOCAL |
| Need of company | *“We all need the spirit of somebody else ... human connectedness ... to bring out our spirituality” (p.71)* | UNEQUIVOCAL |
| Spiritual care | Music and singing was frequently mentioned as uplifting to residents whose memories seemed to embrace familiar songs, particularly hymns. There was repetition of stories about music, prayers, and scripture passages seeming to evoke responses from otherwise non-communicative residents whose disease was well advanced *(p.71)* | UNSUPPORTED. It was based on author’s interpretation |
| Spiritual care | … this included only about 3% of residents with dementia. Most frequently reported approaches to residents with dementia were talking and praying with them and singing hymns (*p. 72, 73)* | UNSUPPORTED |
| Training | Even less (14/74 or 19%) had ever received training for working with residents with dementia. However, the majority thought such care was beneficial (91%) and 65% (48/74) indicated that they would find additional training helpful. *(p.74)* | UNEQUIVOCAL |

**Table S5.9**. Findings from Schmidt et al. [6]

| **FINDINGS** | **ILLUSTRATION** | **EVIDENCE** |
| --- | --- | --- |
| Need of familiarity | *“What I also consider important are stable relationships and stable structures” (p.663)* | UNEQUIVOCAL |

**Table S5.10.** Findings from Stacpoole et al. [25]

| **FINDINGS** | **ILLUSTRATION** | **EVIDENCE** |
| --- | --- | --- |
| Desire of company | *“After she said, ‘Please can you stay with me’, I said, ‘Sorry I*  *can’t stay with you, we have other patients and things to do’.* *She needs us to be there for her, the time wasn’t there for us” (p.332)* | UNEQUIVOCAL |
| Effects of music | *“When she was near to the end, you know, we took her to her room and brought the music box into the room. I remember she used to like Nat King Cole. And I had put that music on… she was very near to the end… and she held up her head, opened her eyes and smiled…” (p. 334)* | UNEQUIVOCAL |
| Namaste Care Effects | *“They are lighting up and they look well. To look at them,*  *they really look well” (p. 335)* | CREDIBLE |
| Namaste Care Effects | *“And you get that relationship… touch builds a relationship between people doesn’t it? …So in that moment there is a really good bond between the staff and the resident” (p.335)* | UNEQUIVOCAL |

**Table S5.11**. Findings from Toivonen et al. [12]

| **FINDINGS** | **ILLUSTRATION** | **EVIDENCE** |
| --- | --- | --- |
| Spiritual music | *The nurse can provide experiences to people with dementia by spiritual music.* *It can make him feel good, reassure him (p.884)* | UNEQUIVOCAL |
| Reminiscence | *“I think that at least the memories of earlier spiritual life are important for people with dementia” (p.884)* | UNEQUIVOCAL |
| Faith community connections | *“For some it is the highlight when the priest comes to visit” (p.885)*  *“I think that religious group participation offers a sense of togetherness” (p.885)* | UNEQUIVOCAL |

**Table S5.12**. Findings from Van der Steen et al. [7]

| **FINDINGS** | **ILLUSTRATION** | **EVIDENCE** |
| --- | --- | --- |
| Spiritual is neglected | Spiritual care is sometimes still neglected, also regarding needs of patient and family from different cultures or traditions *(p.9)* | UNSUPPORTED. It was based on author’s interpretation |
| Informal care | Spiritual care was often not mentioned until prompted and it was not always addressed as systematically as the accounts of what usual care should entail *(p.9-10)* | UNSUPPORTED. It was based on author’s interpretation |
